# Pain neuroscience education in patients with chronic musculoskeletal pain: an umbrella review

**DOI:** 10.3389/fnins.2023.1272068

**Published:** 2023-11-24

**Authors:** Ferran Cuenca-Martínez, Luis Suso-Martí, Joaquín Calatayud, Francisco José Ferrer-Sargues, Vicente Muñoz-Alarcos, Patricio Alba-Quesada, Gemma Biviá-Roig

**Affiliations:** ^1^Department of Physiotherapy, University of Valencia, Valencia, Spain; ^2^Department of Nursing and Physiotherapy, Universidad Cardenal Herrera CEU, CEU Universities, Valencia, Spain

**Keywords:** chronic musculoskeletal pain, pain education, pain neuroscience education, musculoskeletal pain, umbrella review

## Abstract

**Introduction:**

In recent years, pain neuroscience education (PNE) has been the focus of extensive research in the scientific literature in the field of physical therapy, but the results obtained are controversial and its clinical application remains unclear. The main aim of this umbrella review was to assess the effectiveness of PNE in patients with chronic musculoskeletal pain (CMP).

**Methods:**

We searched systematically in PubMed (Medline), PEDro, EMBASE, CINAHL and PsycINFO. Methodological quality was analyzed using AMSTAR-2 scale and overlapping analysis using GROOVE tool.

**Results:**

16 systematic reviews were included. A qualitative synthesis was performed for the following sets of patients with CMP: overall CMP, chronic spinal pain, patients with fibromyalgia and patients with osteoarthritis. In general terms, it seems that the addition of the PNE-based intervention to other treatments, mostly exercise-based interventions although we might refer to it in terms of a multimodal approach, leads to greater clinical improvements than the multimodal approach alone. We have found this especially in the reduction of the influence of psychosocial variables. However, it seems that studies testing the effectiveness of PNE in isolation, systematic reviews with or without meta-analysis did not show statistically significant improvements overall in terms of pain intensity, disability levels or psychosocial variables.

**Discussion:**

There is a great heterogeneity in the results obtained and the PNE protocols used, a critically low quality in the reviews included and a very high overlap, so there is a need to improve the studies in this field before clinical application.

**Systematic review registration:**

PROSPERO (CRD42022355634).

## Introduction

Chronic Musculoskeletal Pain (CMP) is a major public health concern. Approximately 20% of the adult population suffers from CMP (Goldberg and McGee, 2011), which is one of the leading causes of disability worldwide, according to the Global Burden Disease study (Vos et al., 2012). In addition to reducing the quality of life of those who suffer from this condition (Breivik et al., 2006; Tüzün, 2007), it may also result in a serious socioeconomic burden (Espinoza et al., 2022). CMP is a complex and multifactorial phenomenon (Apkarian and Baliki, 2009). The findings of some studies indicate that these patients develop central sensitization mechanisms that contribute to the chronification of pain (Graven-Nielsen and Arendt-Nielsen, 2010; Adams and Turk, 2018). Furthermore, the perception of pain in patients is influenced by factors such as hypervigilance, catastrophizing, kinesiophobia, and anxiety and depression (Keefe et al., 2004). In this light, treating CMP presents a considerable challenge for health care professionals, who should incorporate biopsychosocial approach that address biological, psychological and social factors into their treatment plan (Turk and Okifuji, 2002; Edwards et al., 2016).

Currently, European guidelines emphasize the importance of patient education as part of the treatment of CMP. Education is a planned experience intended to influence the behavior and knowledge of patients through methods of counseling, teaching, and behavior modification (Engers et al., 2008). Patient education increases patients’ knowledge of their condition and promotes positive pain-related beliefs and behaviors (Robinson et al., 2016). Over the past few years, the field of pain neuroscience education (PNE) has attracted a great deal of attention. The aim of this educational strategy is to provide the patient with a thorough understanding of the neurobiological and neurophysiological processes involved in their pain experience (Nijs et al., 2011; Moseley, 2013). It provides patients with an opportunity to improve their understanding of pain and reconceptualize their ideas about it, thereby changing the negative beliefs they have about pain and their incorrect perceptions of it (Moseley and Butler, 2015). Previous research in PNE has suggested that tis intervention could increase knowledge about pain, produce cognitive changes and also have positive effects on pain intensity, disability, kinesiophobia and catastrophizing, as well as on pain-mediating factors such as hypervigilance, anxiety, attitudes and beliefs (Salazar-Méndez et al., 2023; Zimney et al., 2023).

In recent years, PNE has been the focus of extensive research in the scientific literature in the field of physical therapy, and several clinical trials and systematic reviews have been conducted to determine its efficacy. Overall, the results are promising, and show that PNE can be an effective intervention in combination with manual therapy or exercise, although in isolation it may have no effect in patients with CMP (Barbari et al., 2021). However, there are some controversies. A mixed-methods systematic review and meta-analysis found no evidence to indicate that PNE results in clinically important changes over control for pain or disability (Watson et al., 2019). In this regard, it has been suggested that PNE may be effective only for some patients, implying individual differences in response to treatment that may influence outcome variables (Watson et al., 2021). Furthermore, Barbari et al. (2021) highlight that, despite the potential of PNE in CMP patients, the application remains a challenge due to the fact that not all patients can understand PNE concepts in the same way, not all patients are equal despite they are labeled as suffering of chronic MSK pain, and not all clinicians have the training and resources necessaries to apply PNE.

Finally, one challenge in the current scientific literature in this area involves the existence of a large number of systematic reviews, but many of them have overlaps in the studies they include, as well as other methodological shortcomings, which can lead to potentially biased clinical recommendations (Ioannidis, 2016). For this reason, conducting an umbrella review offers a solution, synthesizing the results and conclusions of systematic reviews, and offering insight into possible biases (Lunny et al., 2021). Therefore, the main aim of this umbrella review was to assess and synthetize the previous systematic reviews in the field of PNE for patients with CMP, critically evaluate the published literature in order to elucidate the controversies and determine the effectiveness of PNE in this population.

## Methods

This study was conducted in accordance with the Preferred Reporting Items for Overviews of Systematic Reviews including harm checklist (PRIO-harms), which consists of 27 items (56 sub-items), followed by a 5-stage process flow diagram (identification, screening, eligibility, inclusion, and separation of relevant studies) (Bougioukas et al., 2018). The review was previously registered in the international prospective register of systematic reviews PROSPERO (CRD42022355634).

### Review inclusion criteria

The inclusion criteria employed in this article were based on methodological and clinical factors such as population, intervention, control, outcomes and study design (PICOS) (Stone, 2002).

#### Population

The participants selected for the articles were adults with CMP (including chronic low back or neck pain, osteoarthritis, or rheumatoid arthritis, in addition to those who suffer nonspecific or widespread musculoskeletal pain conditions like fibromyalgia). The diagnosis of CMP was consistent with the British Pain Society definition (chronic pain, that lasts beyond the time that tissue healing would normally be expected to have occurred, often taken as ≥3 months) (British Pain Society, 2013).

#### Intervention and control

We included all systematic reviews assessing the effects of PNE. The intervention should have been composed of planned and structured sessions where patients were educated about the basic neurophysiology of pain, trying to reconceptualize their experience so that it would be considered less threatening (Watson et al., 2019). Interventions based on psychological treatment or cognitive behavioral therapy were excluded. Comparator groups could be non-active interventions, waiting list, minimal interventions (relaxation, breathing or educational advice) or no intervention. If any other treatment (such as medication or manual therapy) was included, it had to be applied in the intervention group as well.

#### Outcome measures

The measures used to assess the results and effects were variables related to clinical outcomes (pain intensity, disability, depressive symptoms, anxiety, pain catastrophizing, and fear-related movement).

#### Study design

We selected systematic reviews (with or without a meta-analysis) of randomized controlled clinical trials (RCCTs) or controlled clinical trials (CCTs) and excluded systematic reviews that included RCCTs or CCTs in combination with non-experimental designs. There were no restrictions for any specific language, as recommended by the international criteria (Moher et al., 1998).

### Search strategy

We conducted the search for published scientific articles between 1950 and November 14^th^, 2022, in the following databases: PubMed (Medline), PEDro, EMBASE, CINAHL, and PsycINFO. The reference sections of the included studies and original studies were screened manually. Supplementary material S1 shows the search strategies, which was adapted for each database. The search was conducted by two independent reviewers (FCM and LSM) using the same methodology. Differences that emerged during this phase were resolved by consensus. The reference sections of the original studies were screened manually, and the authors were contacted for further information if necessary.

### Selection criteria and data extraction

Initially, the two independent reviewers conducted a screening (FCM and LSM) assessing the relevance of the systematic reviews (with and without a meta-analysis) regarding the studies’ questions and objectives. The first screening was based on each study’s title information, abstract, and keywords. The full text was reviewed if there was no consensus or if the abstracts contained insufficient information. In the second phase of the screening, the full text was assessed if the studies met all of the inclusion criteria. Differences between the reviewers were resolved by a discussion and consensus process mediated by a third reviewer (JFFS). The data described in the results section were extracted by means of a structured protocol that ensured that the most relevant information was obtained from each study.

### Methodological quality assessment

The two independent reviewers (FCM and LSM) assessed the methodological quality of the systematic reviews (with or without meta-analysis), assessing each of the selected studies based on the Modified Quality Assessment Scale for Systematic Reviews (AMSTAR-2) developed by Barton et al. (2008) a scale shown to be a valid and reliable tool for assessing the methodological quality of systematic reviews. With a total of 16 items (scoring “yes”; “yes in part” and “no”), with a final assessment as a critically low, low, moderate, and high quality. In addition, we calculated the kappa coefficient (κ) and percentage (%) agreement scores to assess reliability prior to any consensus and estimated the inter-rater reliability using κ: (1) κ > 0.7 indicates a high level of agreement between the reviewers; (2) κ of 0.5–0.7 indicates a moderate level of agreement; and (3) κ < 0.5 indicates a low level of agreement (McHugh, 2012). Disagreements on the final quality assessment score were resolved by consensus with a third independent reviewer (JFFS).

### Overlapping analysis

To assess the overlap of primary studies among systematics reviews the methodological approach GROOVE (Graphical Representation of Overlap for OVErviews) was employed. This tool is an Excel-based file which automatically calculates the overall covered areas for a whole matrix of evidence, and, at the same time, for each possible pair of SRs included in the overview. The tool summarizes the number of reviews, index publications and primary studies (including double counting) included in the matrix. With this data, it calculates the covered area and provides the interpretation of the overall overlap assessment, being slight if the covered area is <5%, moderate if it is ≥5% and < 10%, high if it is ≥10% and < 15%, and very high if is ≥15%. This tool is intended to be used mainly by authors of overviews of systematic reviews (Pérez-Bracchiglione et al., 2022).

## Results

### Study selection

**Figure 1:**
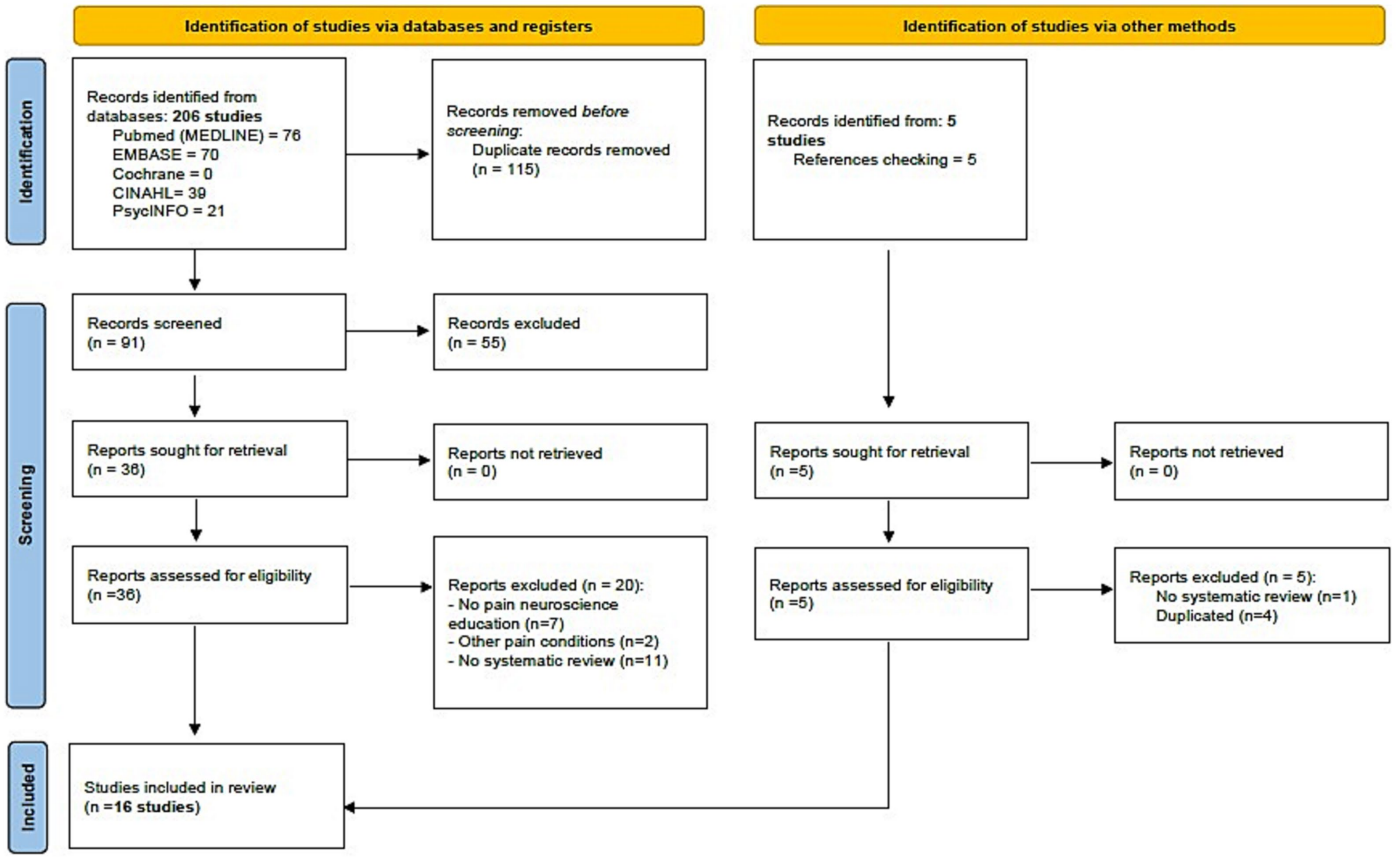
Study flow diagram (PRISMA 2020).

The initial search revealed 206 records, and an additional 5 were retrieved manually from the reference list. Through the title and abstract screening and the full-text assessment, 16 systematic reviews were eligible according to our criteria. The study screening strategy is shown in the form of a flow chart (Figure 1).

### Characteristics of the included systematic reviews

Table 1 lists the characteristics of the systematic reviews included (study design, original studies included, demographic characteristics, interventions, variables, and results). Interventions included PNE combined usually with physical therapy interventions or exercise interventions. The most analyzed tools for pain intensity were the visual analog pain scale (VAS) and numerical pain rating scale (NPRS). For functionality, the RMDQ, neck disability index (NDI), pain disability index (PDI), fibromyalgia impact questionnaire (FIQ), western Ontario and McMaster Universities Arthritis index (WOMAC) and Quebec back pain disability scale (QBPDS) were used. Finally, for the psychosocial variables were the Tampa Scale of Kinesiophobia (TSK) and the Pain Catastrophizing Scale (PCS).

**Table 1:** Characteristics of the included studies.

Study	Studies k (n) Types	Meta-Analysis (k)	Population	Intervention	Control	Outcomes	Results and author’s conclusions
Overall chronic musculoskeletal pain							
Watson et al. (2019)	17 (15867) RCT	Yes (8)	Chronic musculoskeletal pain	PNE traditional physical therapy (manual therapy, exercise therapy)	Traditional physical therapy (manual therapy, exercise therapy) and nonactive interventions, minimal interventions (e.g., relaxation, breathing, or educational advice)	- Pain intensity - Disability - Pain Catastrophizing - Kinesiophobia	The meta-analyzed pooled treatment effects for PNE versus control had low clinical relevance in the short term for pain and disability and in the medium term for pain and disability The treatment effect of PNE for kinesiophobia was clinically relevant in the short term and for pain catastrophizing in the medium term. Low certainty evidence.
Siddall et al. (2022)	5 (263) RCT	Yes (5)	Chronic musculoskeletal pain	PNE combined with an exercise program.	Exercise program alone	- Pain intensity - Disability - Kinesiophobia - Pain catastrophizing	Meta-analysis revealed a significant difference in pain (WMD, 22.09; low certainty), disability (SMD, 20.68; low certainty), kinesiophobia (SMD, 21.20; moderate certainty), and pain catastrophizing (WMD, 27.72; very low certainty) that favored the combination of PNE and exercise. These findings suggest that combining PNE and exercise in the management of chronic musculoskeletal pain results in greater short-term improvements in pain, disability, kinesiophobia, and pain catastrophizing relative to exercise alone.
Marris et al. (2021)	14 (1024) RCT	Yes (8)	Chronic musculoskeletal pain	PNE + traditional physical therapy. Session schedules varied across studies ranging from 1–2 sessions over 4–12 weeks.	- No intervention - Wait-list control - Solely traditional physical therapy	- Pain - Disability	PNE in addition to traditional physical therapy interventions was more effective than traditional physical therapy only, wait-list, or medical management control groups. Meta-analysis results show statistically significant changes in both short and long-term pain and disability with a large effect for both short-term pain intensity and long-term disability. Certainty of evidence N/A.
Romm et al. (2021)	18 (1982) RCT	Yes (8)	Chronic musculoskeletal pain	PNE as a part of intervention	Nonintervention, education, exercise therapy or multimodal physiotherapy	- Kinesiophobia - Pain intensity - Pain disability - Pain catastrophizing	Significant effects of PNE were found on all the outcome measures [pain intensity −0.85 (0.30); disability −0.48 (0.30); kinesiophobia −1.71 (0.36); catastrophizing −0.72 (0.20)]. Certainty of evidence N/A.
Cuenda-gago and Espejo-antúnez (2017)	10 (793) RCT	No	Chronic musculoskeletal pain	PNE in isolation or combination with traditional physical therapy (manual therapy, exercise therapy)	Traditional physical therapy (manual therapy, exercise therapy)	- Pain - Disability - Pain Catastrophizing - Kinesiophobia	PNE alone is effective alone is effective in the short term for the relief of catastrophic pain from catastrophic pain due to erroneous beliefs and attitudes about pain. When PNE is combined with multimodal physiotherapy treatments, it appears that benefits in primary variables (pain and disability) and in some secondary variables (knowledge neurophysiology of pain, catastrophism, pain beliefs and cognitions, kinesiophobia beliefs and cognitions of pain, kinesiophobia, quality of life or life or algometry) are increased. Certainty of evidence N/A.
Kim and Lee (2020)	8 (369) RCT	Yes (8)	Chronic musculoskeletal pain	PNE	Completely different interventions were performed or compared with other educational programs	- Pain - Kinesiophobia	Meta-analysis results showed statistically significant results in favor to PNE compared with control group in pain and kinesiophobia. It was found that PNE alone was more effective when combined with trigger point dry needling and manual therapy. In addition, regardless of therapeutic intensity, a single session alone showed significant improvement, and indirect online education rather than direct education also showed significant improvement. Certainty of evidence N/A.
Louw et al. (2011)	8 (401) RCT	No	Chronic musculoskeletal pain	PNE in isolation or combination with physical therapy	Another treatment, no treatment, or “usual” treatment.	- Pain - Disability - Psychosocial issues	The results of this systematic review show compelling evidence for PNE affecting passive and active physical movements. Positive effects of PNE on pain perception, disability, and catastrophizing may allow patients to apply this new view of their pain state by reappraising their ability to move. Certainty of evidence N/A.
Bülow et al. (2021)	15 (951) RCT	Yes (18)	Chronic musculoskeletal pain	PNE	Conventional therapy or treatment.	- Pain intensity - Disability - Psychological distress (fear avoidance, kinesiophobia, anxiety, catastrophizing, depression).	In chronic musculoskeletal pain, the effects of PNE were moderate and statistically significant on pain intensity and psychological distress at short and long term. Low certainty evidence. However, the effects of PNE on overall were rather small, ranging from −0.93 to −1.16 and − 0.66 to −1.04 for pain intensity and disability on a 0–10 scale.
Louw et al. (2016)	13 (734) RCT	No	Chronic musculoskeletal pain	PNE in isolation or combination with physical therapy	Another treatment, no treatment, or “usual” treatment.	- Pain - Disability - Psychosocial issues	Strong evidence supports the use of PNE for in reducing pain ratings, limited knowledge of pain, disability, pain catastrophizing, fear-avoidance, unhealthy attitudes, and behaviors regarding pain, limited physical movement and healthcare utilization. Certainty of evidence N/A.
Chronic spinal pain							
Bonatesta et al. (2021)	8 (622) RCT	Yes (5)	Chronic Nonspecific Spinal Pain.	PNE + exercise therapy	Nonintervention, education, exercise therapy or multimodal physiotherapy	- Pain intensity - Disability - Kinesiophobia - Catastrophizing	There is low to very-low certainty of the evidence suggesting that PNE plus exercise therapy reduces pain, disability, kinesiophobia, and catastrophizing compared to exercise therapy or multimodal physiotherapy at short- and intermediate-term.
Wood and Hendrick (2019)	8 (6761) RCT	Yes (6)	Chronic low back pain	PNE + exercise therapy, manual therapy, acupuncture, or dry needling	Waitlist controls, physiotherapy, other educational methods, or no treatment.	- Pain - Disability - Psychological effects: Kinesiophobia and Pain catastrophizing	Meta-analysis for short- term pain (n = 428) demonstrated a WMD of 0.73 (95%CI −0.14; 1.61) on a ten-point scale of PNE against no PNE (low certainty evidence). Short-term disability (RMDQ) meta-analysis demonstrated a WMD of 0.42 (moderate certainty evidence); whereas the addition of PNE to physiotherapy interventions demonstrated a WMD of 3.94 (moderate certainty evidence).
Clarke et al. (2011)	2 (122) RCT	No	Chronic Low Back Pain	PNE	Anatomical education	- Pain - Disability - Psychological effects	PNE is a promising intervention for the primary outcome measures of pain, physical-function, psychological- function and social-function. Very low certainty evidence.
Tegner et al. (2018)	7 (1152) RCT	Yes	Chronic Low Back Pain	PNE	No intervention or usual care	- Disability - Pain intensity - Pain catastrophizing - Kinesiophobia	Statistically significant differences in pain, in favor of PNE, were found after treatment, WMD = −1.03 and after 3 months, WMD = −1.09. There was moderate evidence supporting the hypothesis that NPE has a small to moderate effect on pain and low evidence of a small to moderate effect on disability immediately after the intervention. PNE has a small to moderate effect on pain and disability at 3 months follow-up in patients with CLBP
*Osteoarthritis*							
Ordoñez-Mora et al. (2022)	4 (288) RCT and QE	No	Osteoarthritis	PNE	Conventional therapy or treatment.	- Pain intensity - Pain catastrophizing - Kinesiophobia - Disability	Non-pharmacological and educational interventions should be carried out within the interventional processes in patients with pain. The findings revealed an improvement in the groups managed with PNE, finding a small effect in favor of the interventions for variables such as kinesiophobia, with no changes observed in the other variables evaluated. Certainty of evidence N/A.
*Fibromyalgia*							
Suso-Martí et al. (2022)	8 (871) RCT	Yes	Fibromyalgia	PNE	Nonactive interventions, minimal interventions (e.g., relaxation, breathing, or educational advice), or no intervention. If any other treatment (such as medication or manual therapy) was included, it had to be applied in the intervention group as well.	- Pain intensity - FM impact - Anxiety - Pain catastrophizing	Meta-analysis showed statistically significant differences in pain intensity with a moderate clinical effect in seven studies at the post-intervention assessment (SMD: −0.76) but it did not show statistically significant differences in fibromyalgia impact, anxiety, and pain catastrophizing. There is low-certainty evidence that in patients with fibromyalgia, PNE can decrease the pain intensity in the post-intervention period and the fibromyalgia impact in the follow-up period. However, it appears that PNE showed no effect on anxiety and pain catastrophizing.
Saracoglu et al. (2022)	4 (274) RCT	Yes (4)	Fibromyalgia	Multimodal approach including PNE (basic patient education about FM, exercise therapy, cognitive behavioral therapy, mindfulness training and pharmaco- logical treatment)	Treatment as usual, including basic patient education about the disease, recommendations on aerobic exercise, and pharmacological treatment and therapeutic exercise.	- Severity - Pain intensity - Catastrophizing - Depression and anxiety	The meta-analysis showed that PNE groups were statistically more effective on severity of FM (standard mean difference [SMD] = −1.051), pain intensity (SMD = −1.049), catastrophizing (SMD = −0.893), depression (SMD = −0.686) and anxiety (SMD = −0.711). This review demonstrates that adding PNE to a multimodal treatment including exercise therapy might be an effective approach for improving functional status, pain-related symptoms, anxiety, and depression for patients with FM.

CLBP, Chronic Low Back Pain; PNE, Pain Neuroscience Education; RCT, Randomized Control Trial; QE, Quasi-experimental; SMD, Standardized Mean Difference; WMD, Weighted Mean Difference.

The control groups in the studies analyzed by the meta-analyses and reviews included in this paper were: manual therapy (MT), exercise, usual treatment, biomedical/anatomical education, dry needling (DN), self-management techniques.

### Results of the methodological quality (AMSTAR-2)

Table 2 showed the results of methodological quality. All reviews scored as “critically low” quality. The inter-rater reliability of the methodological quality assessment was high (κ = 0.795).

**Table 2:** AMSTAR results.

Study	1	2	3	4	5	6	7	8	9	10	11	12	13	14	15	16	Score
Bonatesta et al. (2021)	Yes	No	No	Partial Yes	Yes	Yes	No	Yes	No	Yes	No	No	Yes	Yes	No	Yes	CL
Bülow et al. (2021)	Yes	Yes	Yes	Partial Yes	Yes	Yes	No	Yes	Yes	No	Yes	No	Yes	Yes	Yes	Yes	CL
Clarke et al. (2011)	Yes	No	Yes	Partial Yes	Yes	Yes	Yes	Yes	Partial Yes	Yes	Yes	Yes	Yes	Yes	Yes	Yes	CL
Cuenda-gago and Espejo-antúnez (2017)	Yes	Partial Yes	Yes	Partial Yes	Yes	Yes	No	Yes	No	No	N/A	N/A	Yes	No	N/A	No	CL
Kim and Lee (2020)	Yes	Yes	Yes	Partial Yes	Yes	Yes	No	Yes	Partial Yes	No	Yes	No	Yes	Yes	Yes	Yes	CL
Louw et al. (2011)	Yes	No	No	Partial Yes	Yes	Yes	No	Yes	No	Yes	N/A	N/A	Yes	Yes	N/A	Yes	CL
Louw et al. (2016)	Yes	No	Yes	Partial Yes	Yes	No	No	Yes	Partial Yes	No	N/A	N/A	Yes	Yes	N/A	Yes	CL
Marris et al. (2021)	Yes	Yes	Yes	Partial Yes	Yes	Yes	No	Yes	Partial Yes	No	Yes	Yes	Yes	Yes	Yes	Yes	CL
Ordoñez-Mora et al. (2022)	Yes	Yes	Yes	Partial Yes	Yes	Yes	Yes	Yes	Yes	No	N/A	N/A	Yes	Yes	N/A	Yes	CL
Romm et al. (2021)	No	Yes	Yes	Partial Yes	Yes	Yes	No	Yes	Yes	No	No	Yes	Yes	Yes	Yes	Yes	CL
Saracoglu et al. (2022)	Yes	Yes	Yes	Partial Yes	Yes	Yes	No	Yes	Yes	No	No	Yes	Yes	Yes	Yes	Yes	CL
Siddall et al. (2022)	No	Yes	Yes	Yes	Yes	Yes	No	Yes	Yes	No	No	Yes	Yes	Yes	Yes	Yes	CL
Suso-Martí et al. (2022)	Yes	Yes	Yes	Partial Yes	Yes	Yes	No	Yes	Yes	No	No	Yes	Yes	Yes	Yes	Yes	CL
Tegner et al. (2018)	Yes	No	Yes	Partial Yes	Yes	Yes	No	Yes	Yes	No	Yes	Yes	Yes	Yes	Yes	Yes	CL
Watson et al. (2019)	Yes	Yes	Yes	Partial Yes	Yes	Yes	Yes	Yes	Yes	No	Yes	Yes	Yes	Yes	Yes	Yes	CL
Wood and Hendrick (2019)	Yes	Yes	Yes	Partial Yes	Yes	Yes	No	Yes	Yes	No	Yes	Yes	Yes	Yes	Yes	Yes	CL

CL, Critically Low. AMSTAR 1: Did the research questions and inclusion criteria for the review include the components of PICO? AMSTAR 2: Did the report of the review contain an explicit statement that the review methods were established prior to the conduct of the review and did the report justify any significant deviations from the protocol? AMSTAR 3: Did the review authors explain their selection of the study designs for inclusion in the review? AMSTAR 4: Did the review authors use a comprehensive literature search strategy? AMSTAR 5: Did the review authors perform study selection in duplicate? AMSTAR 6: Did the review authors perform data extraction in duplicate? AMSTAR 7: Did the review authors provide a list of excluded studies and justify the exclusions? AMSTAR 8: Did the review authors describe the included studies in adequate detail? AMSTAR 9: Did the review authors use a satisfactory technique for assessing the risk of bias in individual studies that were included in the review? AMSTAR 10: Did the review authors report on the sources of funding for the studies included in the review? AMSTAR 11: If meta-analysis was performed did the review authors use appropriate methods for statistical combination of results? AMSTAR 12: If meta-analysis was performed, did the review authors assess the potential impact of risk of bias in individual studies on the results of the meta-analysis or other evidence synthesis? AMSTAR 13: Did the review authors account for risk of bias in individual studies when interpreting/ discussing the results of the review? AMSTAR 14: Did the review authors provide a satisfactory explanation for, and discussion of, any heterogeneity observed in the results of the review? AMSTAR 15: If they performed quantitative synthesis did the review authors carry out an adequate investigation of publication bias (small study bias) and discuss its likely impact on the results of the review? AMSTAR 16: Did the review authors report any potential sources of conflict of interest, including any funding they received for conducting the review.

### Qualitative synthesis

A subgroup analysis was performed with the aim of homogenizing the results. Therefore, a qualitative synthesis was performed for the following sets of patients with CMP: reviews that included all kind of patients with CMP (overall CMP), chronic spinal pain, patients with fibromyalgia and patients with osteoarthritis. In addition, results were presented according to the different time points: short term (< 3 months); medium-term (> 3 months but < 12 months) or long-term (≥ 12 months). A common effect size was estimated using the Cochrane recommendations and used for the qualitative synthesis in the figures (Higgins et al., 2023).

### Overall CMP

#### Pain intensity

Three meta-analyses found between-group differences in pain scales showing an overall moderate effect favorable to PNE [Marris et al. (2021): Standardized Mean Difference (SMD) = −0.756; 95% Confidence Interval (CI): −0.059 to −1.571; Kim and Lee (2020): SMD = −0.53; 95% CI: −1.05 to −0.01; Romm et al. (2021): SMD = −0.85; 95% CI: −1.46 to −0.23].

**Figure 2:**
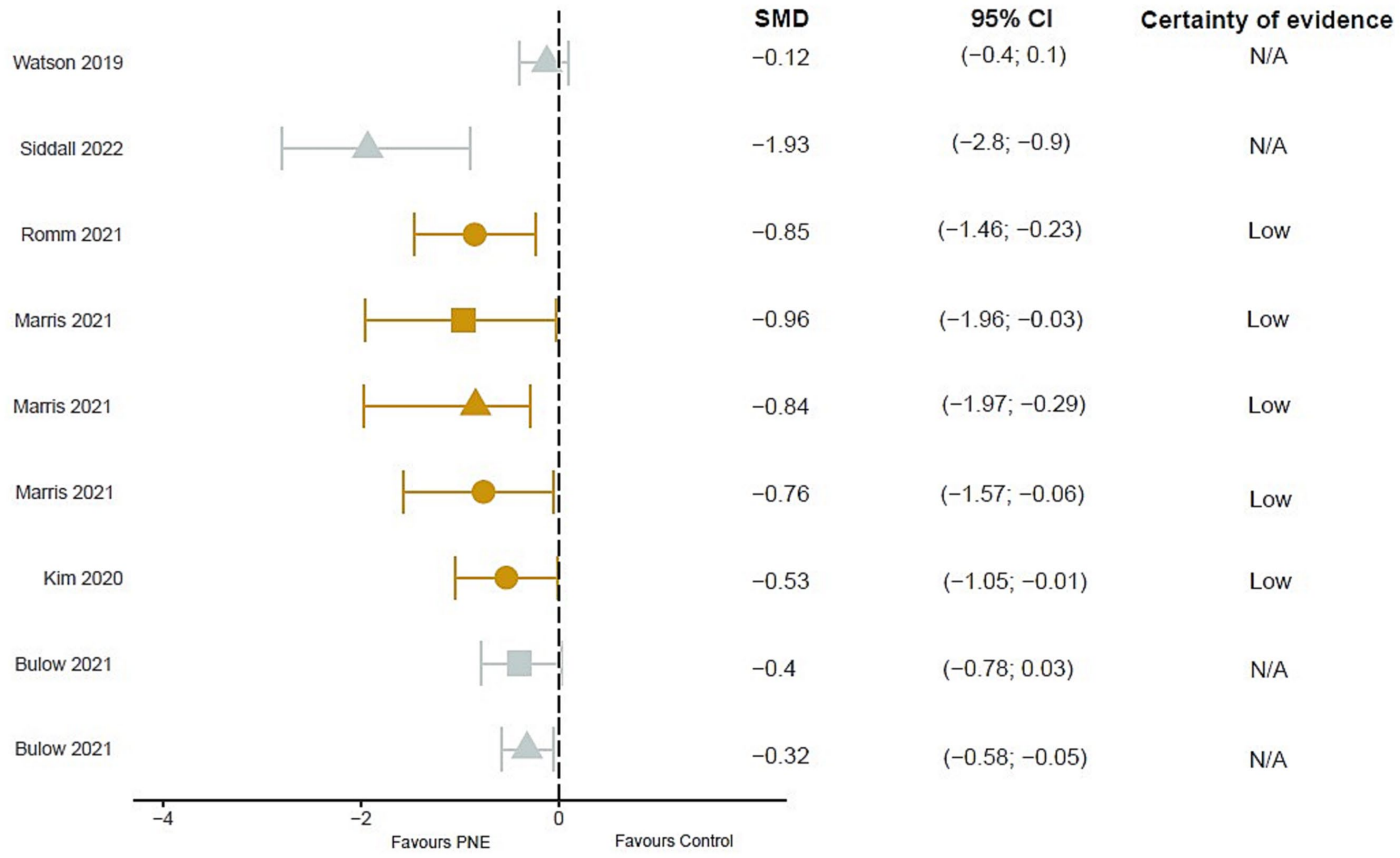
Results of mean differences regarding pain intensity in patients with overall chronic musculoskeletal pain. The colors show the certainty of evidence according to GRADE (red = very low certainty evidence; yellow = low certainty evidence; green = moderate certainty evidence, grey = not available). Circles = overall measurement; Triangles = short term measurement; square = long term measurement. SMD, Standardized mean difference; N/A, Not available; CI, Confidence interval.

In a temporal analysis, 4 meta-analyses observe a low to large effect favorable to PNE in the short term [Bülow et al. (2021): SMD = 0.32; 95% CI: 0.58 to −0.05; Watson et al. (2019): MD = 5.91; 95% CI: −13.75 to 1.93; Marris et al. (2021): SMD = 0.837; 95% CI: −0.299 to 1.972; Siddall et al. (2022): SMD = −2.09; 95% CI: 3.38 to −0.80] and low quality of evidence (Watson et al., 2019). 2 meta-analyses extrapolate the effects of PNE in the long term, showing small to large effects favorable to PNE [Bülow et al. (2021): SMD = 0.40; 95% CI: 0.78 to 0.03; Marris et al. (2021): SMD = 0.964; 95% CI: −0.032 to 1.959] (Figure 2).

Similarly, different reviews indicate that the combined treatment of PNE with exercise or another intervention showed better outcome compared to the conventional physiotherapy control group, a usual medical control, or an exercise-only group in the short, medium and long term (Louw et al., 2011, 2016; Cuenda-gago and Espejo-antúnez, 2017; Barbari et al., 2020).

#### Disability

6 meta-analyses (Watson et al., 2019; Bülow et al., 2021; Marris et al., 2021; Romm et al., 2021; Watson et al., 2021; Siddall et al., 2022) and 3 reviews (Louw et al., 2011, 2016; Cuenda-gago and Espejo-antúnez, 2017) analyzed the effect of PNE on different tools, such as the RMDQ, ODI, FIQ questionnaires and the QBPD and SF-36 scales.

**Figure 3:**
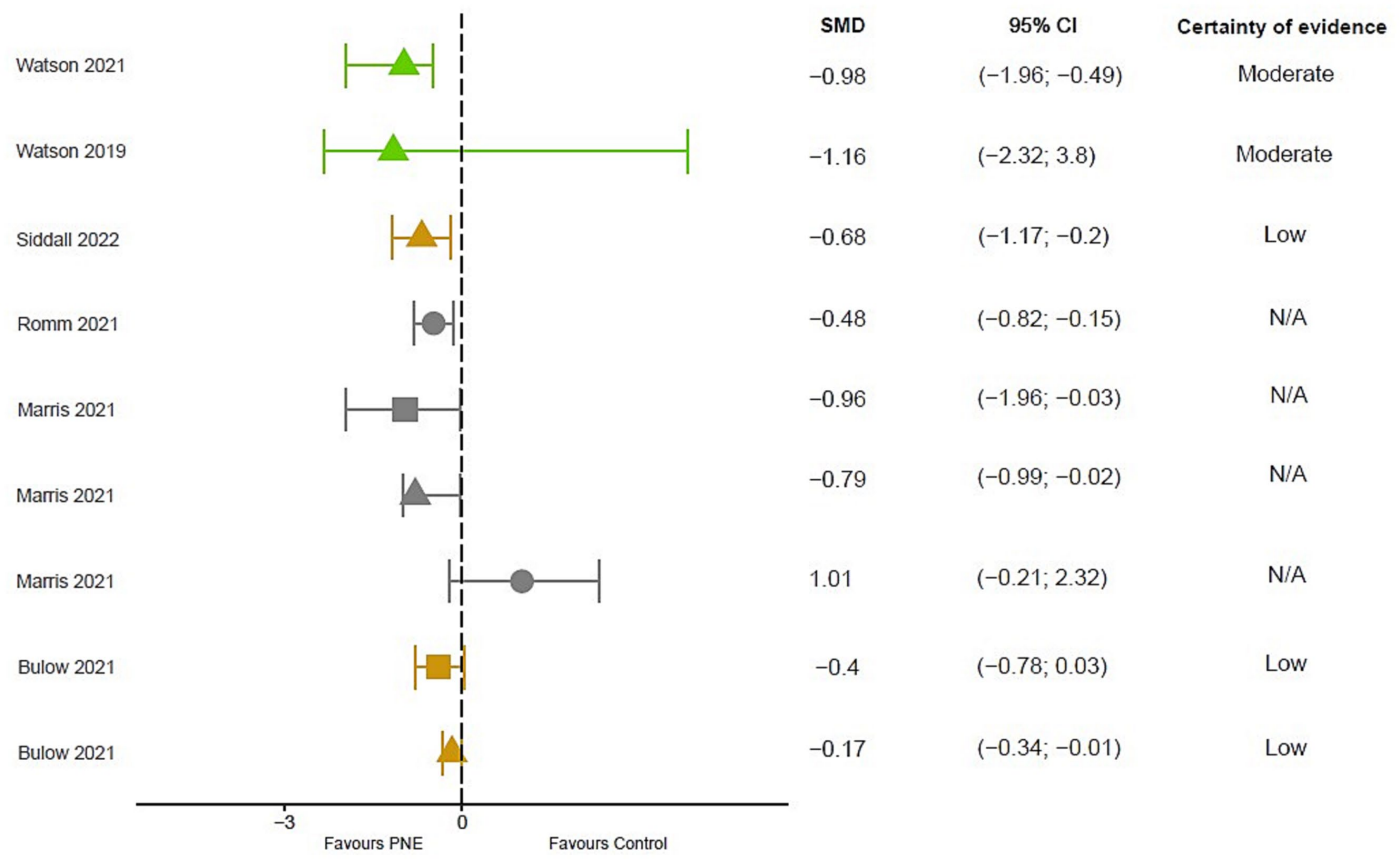
Results of mean differences regarding disability in patients with overall chronic musculoskeletal pain. The colors show the certainty of evidence according to GRADE (red = very low certainty evidence; yellow = low certainty evidence; green = moderate certainty evidence, grey = not available). Circles = overall measurement; Triangles = short term measurement; square = long term measurement. SMD, Standardized mean difference; N/A, Not available; CI, Confidence interval.

2 meta-analyses observe a moderate to large effect in favor of PNE when compared to control groups following self-management education or treatment as usual, but with no statistically significant result in the case of Marris et al. (2021) (SMD = 1.009; 95% CI: −0.213 to 2.232) and showing statistically significant differences in Romm et al. (2021) (SMD = −0.48; 95% CI −0.82 to −0.15). In the short term 5 meta-analyses observed a small to moderate with moderate evidence favorable to PNE [Bülow et al. (2021): SMD = 0.17; 95% CI −0.34 to −0.01; Watson et al. (2019): MD = 4.09, 95% CI = 7.72 to 0.45; Watson et al. (2021): MD = 7.36, 95% CI = 3.93 to 11.12; Marris et al. (2021): SMD = 0.791; 95% CI −0.994 to 2.575; Siddall et al. (2022): SMD = −0.68; 95%CI −1.17 to −0.2]. 3 meta-analyses analyzed the effect in the medium and long term, favorable effects were observed with moderate evidence in the medium term [Watson et al. (2019): MD = 8.14]. In the long term, no statistically significant differences were observed [Bülow et al. (2021): SMD = 0.27; 95% CI: 0.59 to 0.06; Marris et al. (2021): SMD = 1.374; 95% CI: −0.103 to 2.850] (Figure 3).

The included reviews observe superior effects of the use of PNE with respect to the control groups of conventional physical therapy or usual medical follow-up for the tools used RMDQ, ODI, NDI and PSFS when PNE is combined with TM and/or exercise (Louw et al., 2011, 2016; Cuenda-gago and Espejo-antúnez, 2017). However, controversies exist as no superiority could be demonstrated for the combination of PNE and DN compared to DN alone, as both groups improved on the ODI and NDI scale (Louw et al., 2016). A meta-analysis highlights a possible dependence of the control group compared to NSP on the total outcome effects (Romm et al., 2021).

#### Psychosocial variables

5 meta-analyses (Watson et al., 2019; Kim and Lee, 2020; Bülow et al., 2021; Romm et al., 2021; Siddall et al., 2022) and 3 reviews (Louw et al., 2011, 2016; Cuenda-gago and Espejo-antúnez, 2017) conducted an analysis on the effects of PNE on variables such as kinesiophobia or catastrophizing.

4 meta-analyses found a large and statistically significant favorable effect in relation to kinesiophobia (TSK) [Romm et al. (2021): SMD = −1.71; Kim and Lee (2020): SMD = −0.86; 95% CI: −1.22 to −0.51; Watson et al. (2019): MD = 13.55; 95% CI, −25.89 to −1.21; Siddall et al. (2022); SMD = −1.20; 95% CI: 1.84 to −0.57]. In addition, 1 meta-analysis also found a benefit in catastrophizing [Watson et al. (2019): MD = 3.33; 95% CI: −6.01 to −0.65].

The included reviews follow the trend of favorable analysis of the use of PNE in the different treatments for TSK, PCS, SOPAR, and FABS scales (Louw et al., 2011, 2016; Cuenda-gago and Espejo-antúnez, 2017). They also highlight the possible influence of a group and individual treatment and the difference between only receiving one PNE session or several.

#### Overlap analysis

**Figure 4:**
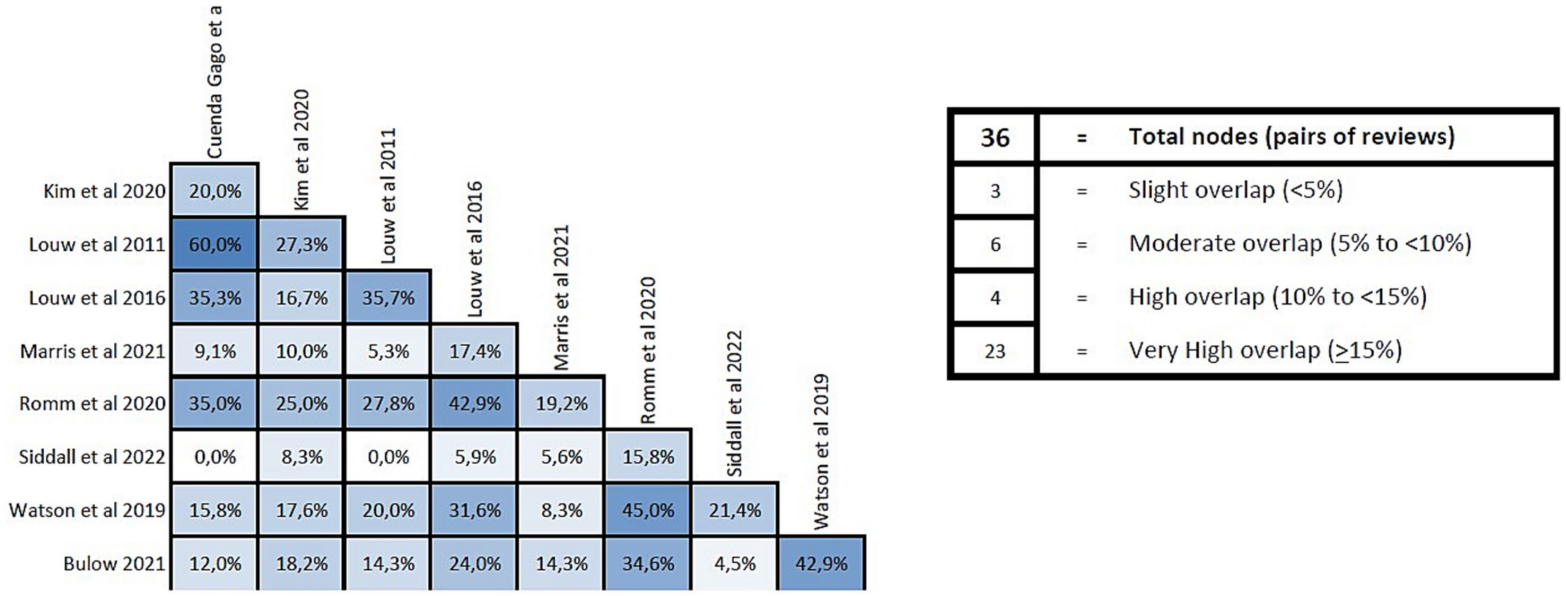
Overlap analysis performed using the GROOVE regarding reviews in patients with chronic musculoskeletal pain.

The overlap analysis performed using the GROOVE tool showed a corrected overlap area of 16.45%, showing a very high level of overlap. The detail of the overlap is shown in Figure 4.

### Chronic spinal pain

#### Pain intensity

**Figure 5:**
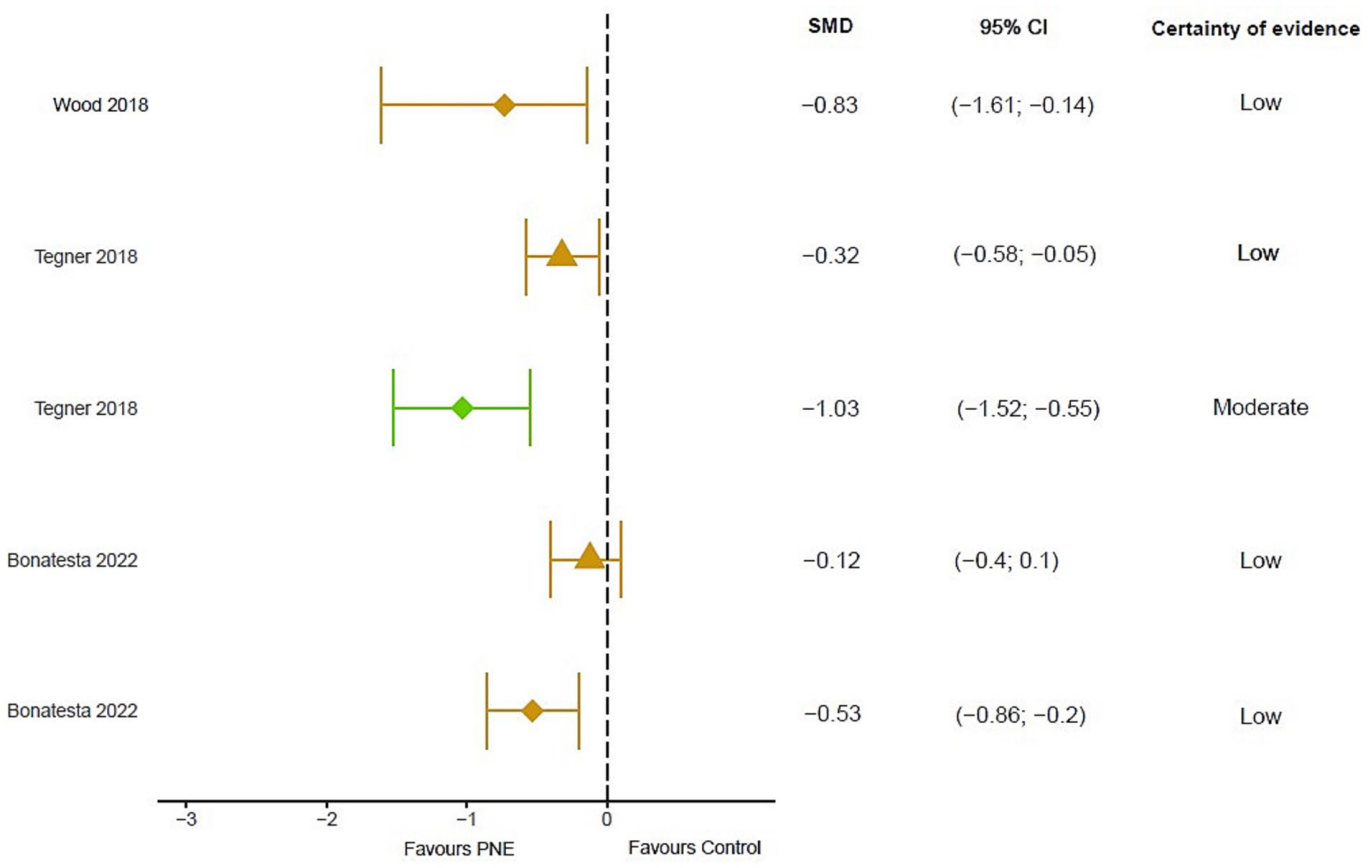
Results of mean differences regarding pain intensity in patients with overall chronic spinal pain. The colors show the certainty of evidence according to GRADE (red = very low certainty evidence; yellow = low certainty evidence; green = moderate certainty evidence, grey = not available). Circles = overall measurement; Triangles = short term measurement; diamond = medium term. SMD, Standardized mean difference; N/A, Not available; CI, Confidence interval.

2 meta-analyses observed a small effect favorable to PNE with low evidence in the short term for low back pain [Wood and Hendrick (2019): Weighted Mean Difference (WMD) = 0.73; 95% CI −0.14 to 1.61; Tegner et al. (2018): WMD = −1.03; 95% CI: −0.55 to −1. 52], found similar results in general spine pain [Bonatesta et al. (2021): SMD = −0.53; 95% CI: −0.86 to −0.20]. In the medium term 2 meta-analyses showed similar results [Tegner et al. (2018): WMD = −1.09; 95% CI: −2.17 to 0.00; Bonatesta et al. (2021): SMD = −0.57; 95% CI: −1.01 to −0.14] (Figure 5).

2 meta-analyses analyzed the effects of PNE as sole treatment in low back pain, where a statistically significant result was obtained in the short term [Clarke et al. (2011): MD = 5] although these changes were not clinically significant. In contrast, in the medium term, significant clinical changes favorable to PNE were observed [Clarke et al. (2011): MD = 19]. The addition of PNE to treatment appears to improve outcomes in pain intensity, maintained in the short and long term (Barbari et al., 2020).

#### Disability

**Figure 6:**
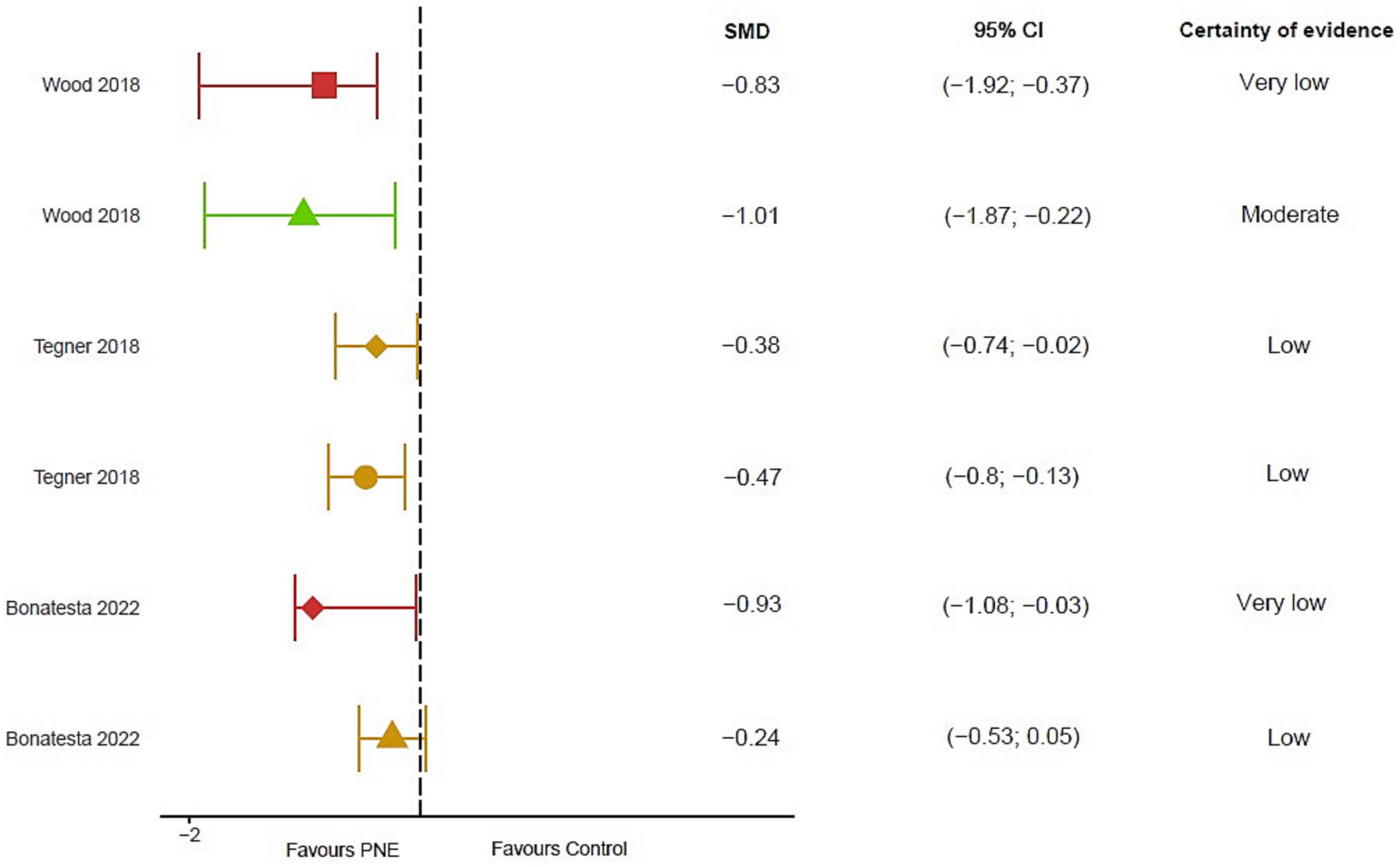
Results of mean differences regarding disability in patients with overall chronic spinal pain. The colors show the certainty of evidence according to GRADE (red = very low certainty evidence; yellow = low certainty evidence; green = moderate certainty evidence, grey = not available). Circles = overall measurement; Triangles = short term measurement; square = long term measurement; diamond = medium term. SMD, Standardized mean difference; N/A, Not available; CI, Confidence interval.

In the short term 2 meta-analyses observed a clinically significant effect of PNE together with multimodal treatment with moderate quality evidence [Wood and Hendrick (2019): WMD = 3.94; 95% CI 3.37 to 4.52; Tegner et al. (2018): SMD = −0.47; 95% CI: −0.80 to −0.13]. However, 1 meta-analysis observed no differences in the short-term use of PNE combined with exercise compared with the exercise-only control group with very low-quality evidence [Bonatesta et al. (2021): SMD = −0.24; 95% CI: −0.53 to 0.05]. 2 meta-analyses found favorable differences for PNE compared with exercise alone in the medium-term [Bonatesta et al. (2021): SMD = −0.93; 95% CI: −1.08 to −0.03; Tegner et al. (2018): SMD = −0.38; 95% CI, −0.74 to −0.02] with a low to moderate effect and very low-quality evidence. These results were not maintained in the long term [Wood and Hendrick (2019): WMD = 2.18; 95% CI: −0.67 to 5.02] (Figure 6).

#### Psychosocial variables

2 meta-analyses demonstrated a positive but not statistically significant effect for kinesiophobia in the short term [Wood and Hendrick (2019): WMD = 4.72; 95% CI: 2.32 to 7.13; Tegner et al. (2018): WMD = −5.73 (95% CI: −13.06 to 2.14)] or a global spine analysis [Bonatesta et al. (2021): SMD = −0.70; 95% CI: −1.51 to 0.11].

Similarly, 2 showed a positive but not statistically significant effect for catastrophizing in the short term for low back pain [Wood and Hendrick (2019): WMD = 2.54; 95% CI: −4.23 to 9.31] or the overall spine [Bonatesta et al. (2021): MD = −3.26; 95% CI: −6.15 to −0.37].

#### Overlap analysis

**Figure 7:**
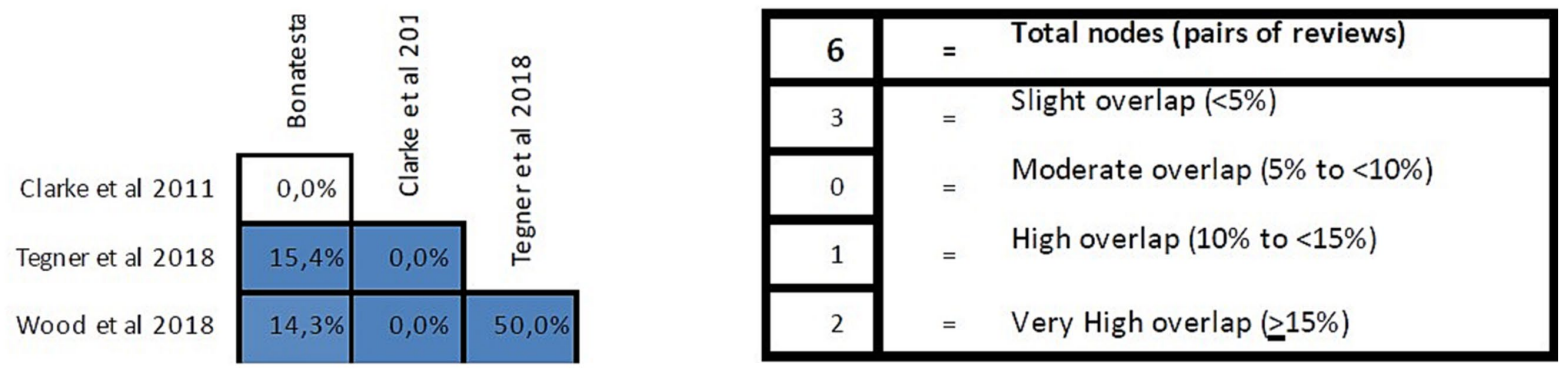
Overlap analysis performed using the GROOVE regarding reviews in patients with chronic spinal pain.

The overlap analysis performed using the GROOVE tool showed a corrected overlap area of 15.45%, showing a very high level of overlap. The detail of the overlap is shown in Figure 7.

### Fibromyalgia

Two reviews with meta-analysis (Saracoglu et al., 2022; Suso-Martí et al., 2022) and two reviews analyzed the total effect of PNE in fibromyalgia. The most analyzed tools for pain intensity were the VAS scale and NPRS, and for assessing functionality the FIQ questionnaire and a subscale of the ICF. As for psychosocial variables, they were the HADS scale and the PCS questionnaire. The control groups with which the PNE was compared in the studies analyzed by the meta-analyses and reviews included in this work were: self-management techniques, TM, no intervention, exercise, other educational pathways.

Two meta-analyses evaluated the effect of PNE on pain intensity, showing significant differences between the intervention groups compared to the control group, where PNE obtained a moderate [Suso-Martí et al. (2022): SMD: −0.76; 95% CI: −1.33 to −0.19] to large [Saracoglu et al. (2022): SMD = −1.049; 95% CI = −1.400, −0.698] effect for the variables VAS and NPRS.

In the medium- and long-term analysis, it could not be affirmed (without significant differences) that PNE decreases pain intensity [Suso-Martí et al. (2022): SMD: −0.42; 95% CI: −0.93 to 0.08], although an overall favorable effect can be observed. PNE improved the efficacy of endogenous pain mechanisms and active coping to persistent pain (García-Ríos et al., 2019). In contrast, a review could not conclude that PNE in isolation treatment has a positive effect on pain intensity in the short and long term (Elizagaray-garcía et al., 2016).

The impact of fibromyalgia was measured by the FIQ questionnaire, where a meta-analysis did not observe a significant difference for PNE in post-intervention [Suso-Martí et al. (2022): SMD: −0.37; 95% CI: −0.85 to 0.11], also observing contradictory evidence on the CIF and SF-36 functionality subscale in the short-medium term where the combination of exercise and group PNE or other active coping strategies did improve pain intensity for the CIF subscale (Elizagaray-garcía et al., 2016). We did observe a favorable clinical effect on FM severity in the medium and long term in PNE [Suso-Martí et al. (2022): SMD = 0.44; 95% CI: −0.73 to −0.14] coinciding with another meta-analysis showing a large effect of PNE in the intervention groups (SMD = −1.051; 95% CI: −1.309 to −0.793) (Saracoglu et al., 2022).

Regarding psychosocial variables, the meta-analysis by Suso-Martí et al. (2022) evaluated PNE outcomes in the short term, where no significant difference was observed for anxiety (SMD = −0.06; 95% CI: −0.67 to 0.55) and catastrophizing (SMD = −0.10; 95% CI: −0.52 to 0.32). In the medium and long term, no statistical differences were also found for anxiety (SMD = −0.07; 95% CI: −0.69 to 0.82) or catastrophizing (SMD = −0.16; 95% CI: −0.52 to 0.19). However, the Saracoglu et al. (2022) meta-analysis did note statistical differences between groups favorable to PNE for the PCS scale with a large effect (SMD = −0.893; 95% CI: −1.437 to −0.348). A moderate effect was also noted for the HADS scale for anxiety (SMD = −0.711; 95% CI: −0.869 to −0.552) and depression (SMD = −0.686; 95% CI: −0.849 to −0.523).

### Osteoarthritis

Only one review analyzed the effects of PNE as a treatment in intervention groups (Ordoñez-Mora et al., 2022). Only 4 studies were included in the review and were performed on patients with radiologically proven knee OA (with diagnosis time of over 6 months) and candidates for total knee arthroplasty. Regarding pain intensity, measured by the NRS scale, a positive trend in the use of PNE was observed, but without significant differences when compared to a group without intervention or preoperative biomedical education with manual therapy as a common treatment. However, there were significant changes in favor of PNE combined with exercise compared to exercise alone. There were no significant differences between groups for functionality in the WOMAC scale analysis when PNE was compared to biomedical education. For psychosocial variables, an overall favorable trend was indicated for TSK and PCS without being significant.

## Discussion

The main aim of this umbrella review was to assess the effectiveness of PNE in patients with CMP. In general terms, it seems that the addition of the PNE-based intervention to other treatments, mostly exercise-based interventions although we might refer to it in terms of a multimodal approach, leads to greater clinical improvements than the multimodal approach alone. We have found this especially in the reduction of the influence of psychosocial variables. However, it seems that studies testing the effectiveness of PNE in isolation, systematic reviews with or without meta-analysis did not show statistically significant improvements overall in terms of pain intensity, disability levels or psychosocial variables. It seems therefore that the main strength of the PNE is the interaction with other interventions to enhance its effectiveness with respect to the outcomes assessed. All the reviews included had scored as “critically low” quality and the overlapping analysis showed a very high level of overlap. These aspects limit the clinical application of the recommendations made to date on PNE in CMP patients, and therefore its clinical efficacy is uncertain and dependent on several factors that need to be further explored.

PNE is a clinical intervention that has the communication process as a key point of its application and where the patient feels listened to, cared for and, in addition, allows patients to better understand their clinical condition process (Street et al., 2009). This increased knowledge from a patient perspective, together with an adequate context promoted by empathy, shared understanding between health professional and patient and increasing social support, seems to help improve the influence of psychological variables that are widely present in chronic musculoskeletal pain processes (Lee et al., 2016; Traeger et al., 2019). However, the mechanisms that explain the functioning and benefits of PNE are not entirely clear. It has been suggested that this intervention might be especially relevant for the affective-emotional component of pain (Moseley and Butler, 2015), which could lead to better coping strategies (Nijs et al., 2017). In addition, PNE could modify perceptual error in threat signal assessment in the neural network involved in pain perception, reducing nervous system sensitivity and brain activity (Saracoglu et al., 2022).

Based on the results of the present review, a clinical approach based solely on PNE alone may be insufficient to provide clinically relevant and meaningful results, and it seems that the current state of the art tells us that we should combine it with an active and/or passive intervention (such as exercise-based interventions, manual neuro-orthopedic physical therapy, etc.) in order to improve its effectiveness. Positive effects on decreasing pain intensity, disability levels or catastrophic thoughts have been reported when researchers combined PNE together with an exercise-based intervention compared with the exercise-based intervention in isolation in patients with chronic musculoskeletal pain (Siddall et al., 2022) or in patients with chronic non-specific spinal pain (Bonatesta et al., 2021). Given that exercise has already shown positive results in patients with musculoskeletal disorders with pain such as fibromyalgia syndrome (Sosa-Reina et al., 2017; Estévez-López et al., 2021) or chronic non-specific low back pain (Hayden et al., 2005; Searle et al., 2015) in the scientific literature, future studies seem to address whether PNE could improve the efficacy of exercise-based interventions, as is the case for chronic musculoskeletal pain. It seems that our work sheds some light on this issue, although future studies should address some of the limitations and weaknesses found in the present manuscript. Finally, there is great variability in the results found depending on the different variables (content of the sessions, the format, the interventions added to the session, the characteristics of the population or the duration of the intervention). In this regard, recently, a meta-analysis found a linear association between the duration of PNE and the reduction of anxiety symptoms, catastrophizing and kinesiophobia (Salazar-Méndez et al., 2023). Future studies should determine not only whether PNE is effective, but also in which patients it is effective and in what way it is best to apply it.

## Limitations

This study presents several limitations. First, part of the included studies presented low methodological quality and a great heterogeneity. Second, there was inconsistency between the systematic reviews in terms of the interventions and control groups, and this limits the strength of the conclusions. Finally, as no statistical aggregation could be performed due to the low number of included studies, the conclusions are somewhat ambiguous as they satisfy a qualitative analysis, and not a quantitative one (which would be more robust).

## Conclusion

The addition PNE-based intervention to other treatments leads to greater clinical improvements than alone interventions based on physical therapy or exercise modalities, especially in the reduction of the influence of psychosocial variables. However, the effectiveness of PNE in isolation did not show statistically significant improvements overall in terms of pain intensity, disability levels or psychosocial variables. There is a great heterogeneity in the results obtained and the PNE protocols used, a critically low quality in the reviews included and a very high overlap, so there is a need to improve the studies in this field before clinical application.

## Data availability statement

The original contributions presented in the study are included in the article/Supplementary material, further inquiries can be directed to the corresponding author.

## Author contributions

FC-M: Investigation, Methodology, Writing – original draft, Writing – review & editing. LS-M: Conceptualization, Data curation, Methodology, Resources, Supervision, Validation, Writing – original draft, Writing – review & editing. JC: Data curation, Formal Analysis, Project administration, Writing – original draft, Writing – review & editing. FF-S: Investigation, Resources, Supervision, Visualization, Writing – original draft, Writing – review & editing. VM-A: Methodology, Writing – original draft, Writing – review & editing. PA-Q: Methodology, Writing – original draft, Writing – review & editing. GB-R: Conceptualization, Data curation, Formal Analysis, Investigation, Project administration, Resources, Writing – original draft, Writing – review & editing.
