# Supplementary material for: Pain neuroscience education in patients with chronic musculoskeletal pain: an umbrella review
Source: Front Neurosci. 2023 Nov 24;17:1272068. doi: 10.3389/fnins.2023.1272068 (PMC10704151; doi:10.3389/fnins.2023.1272068)
Supplement: Supplementary file 1 [file Data_Sheet_1.docx]

**Supplementary material 1.** Database search equations

***Medline (PubMed)***

("Pain Neuroscience Education"[All Fields] OR "Pain Education"[All Fields] OR "Pain Neurophysiology Education"[All Fields] OR "Pain Psychology"[All Fields]) OR "Psychoeducational Intervention"[All Fields] OR "Health Education"[All Fields] OR "Pain Physiology Education"[All Fields]) OR "Therapeutic Neuroscience Education"[All Fields] OR "Pain Biology"[All Fields] OR "Cognitive Education"[All Fields] OR "Therapeutic Patient Education"[All Fields] OR "Explain Pain"[All Fields] OR "Therapeutic Neurobiology"[All Fields] AND ("pain"[All Fields] OR "pain intensity"[All Fields] OR ("pain"[MeSH Terms] OR "pain perception"[MeSH Terms] OR "pain threshold"[MeSH Terms] OR "pain measurement"[MeSH Terms] OR “visual analogue scale” [MeSH Terms] OR “disability”[MeSH Terms] AND “Chronic Pain”[MeSH Terms] OR “Persistent Pain”[All Fields] OR “Persistent Pain”[All Fields] OR “Musculoskeletal Pain”[All Fields] AND “Review” [All Fields] OR “Systematic Review”[All Fields]
